# Effect of stevia leaves (*Stevia rebaudiana* Bertoni) on diabetes: A systematic review and meta‐analysis of preclinical studies

**DOI:** 10.1002/fsn3.2904

**Published:** 2022-04-24

**Authors:** Akibul Islam Chowdhury, Mohammad Rahanur Alam, M Maruf Raihan, Tanjina Rahman, Saiful Islam, Oumma Halima

**Affiliations:** ^1^ 378872 Department of Food Technology and Nutrition Science Noakhali Science and Technology University Noakhali Bangladesh; ^2^ 378872 Institute of Nutrition and Food Science University of Dhaka Dhaka Bangladesh

**Keywords:** animal study, blood sugar, diabetes, *Stevia rebaudiana*, systematic review

## Abstract

Stevia (*Stevia rebaudiana* Bertoni) is a natural herb with biological activities such as anticancer, antidiabetic, anticardiovascular disease, anti‐inflammatory, and antimicrobial. The current systematic review and meta‐analysis of previously published data were performed to assess the antidiabetic effect of stevia leaves. Three electronic databases (PubMed, CENTRAL, and DOAJ) had been used for searching articles published before September 2020. Meta‐analysis via random‐effect model had been performed to assess the effects of different doses of stevia on blood glucose level (BGL) and studies were weighted according to an estimate of the standard mean difference (SMD). Overall, 16 eligible studies were selected for qualitative analysis and 9 were included for quantitative analysis. The results of the meta‐analysis for BGL showed that at the doses of 200, 300, and 400 mg/kg of stevia leaves there was a significant difference in means of BGL between the intervention and control group and the dose of 500 mg/kg showed no significance (Standard mean difference (*SMD*): −3.84 (−9.96, 2.27); *p* = .22). Based on the duration of intervention, subgroup analysis of articles showed a significant difference between the groups (*p* < .001). The results of the meta‐analysis support the hypothesis that stevia leaf has an antihyperglycemic effect and reduces the blood glucose level at doses of 200, 300, and 400 mg/kg. Therefore, more clinical trials on animals and humans have to be done to investigate the antidiabetic and antihyperglycemic effects along with the efficacy and safety of these medicinal leaves.

## Graphical abstract

*Stevia rebaudiana* Bertoni has antidiabetic and antihyperglcemic activities presented in many studies. The present systematic review and meta‐analysis showed *S*. *rebaudiana* positively reduce blood glucose level. Significant dose‐dependent relationship of intervention also showed among the groups in subgroup analysis. However, higher dose (500 mg/kg) did not show significant result in meta‐analysis (*p* > .05). Duration of intervention was also significantly associated with reducing diabetes (*p* < .01).

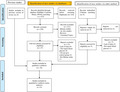

## 1 INTRODUCTION

Diabetes is a common metabolic disease resulting from insulin deficiency or insulin resistance at the cellular level. It is associated with several noncommunicable diseases (NCDs), such as dysfunction or failure of organs, like kidneys, heart, blood vessel, thus predisposed to hypertension, renal disease, ocular diseases, stroke, heart failure, obesity, etc. (Mellitus, 2005). Among other types of diabetes, it is estimated that around 90–95% of patients with diabetes are suffering from Type‐2‐diabetes (T2D) in the world (Issa & Hussen Bule, 2015). Several markers are used to identify T2D among patients, such as glucose intolerance, fasting blood glucose (FBG), insulin secretion level, serum glucose level (SGL) (Rahmani et al., 2018). Management of diabetes is complex and needs to follow several strategies such as consumption of balanced diet, knowledge of diet‐related factors that may help in controlling blood glucose level (BGL), physical exercise, and, if needed, proper use of insulin. Natural antihyperglycemic agents are used nowadays to treat diabetes (Gaudel et al., 2013; Milani et al., 2016). Natural agents having antihyperglycemic activity are considered to be safe, less toxic, and inexpensive compared with pharmacological drugs which are used to treat diabetic patients. Along with these benefits, natural agents like stevia leaves have fewer adverse side effects than these drugs (Dhasarathan & Theriappan, 2011; Li et al., 2008). *Stevia rebaudiana* (Bertoni), also called sweet tulsi leaf, is a kind of antihyperglycemic plant that has been used as a safe sweetening agent over many decades (Barriocanal et al., 2008).

*Stevia rebaudiana* (Bertoni) belonging to the aster or chrysanthemum family contains eight sweet diterpene glycosides, protein, fibers, carbohydrates, phosphorus, iron, calcium, potassium, flavonoids (rutin), zinc, vitamin A, and vitamin C. Glycoside is the main component of stevia that produces sweet taste without having no‐calorie (Kim et al., 2002). The US Food and Drug Administration (FDA) approved Rebaudioside as a safe sweetener and supplement (Administration, 2008). As stevioside, a major component of stevia is 200–300 times sweeter than sucrose. It is cultivated in many parts of Brazil, China, Thailand, Paraguay, Central America, and India commercially and widely used in foods, beverages, medicine, cosmetics, and many other food industries (Stoyanova et al., 2011). Stevia is used for treating several kinds of noncommunicable diseases (NCDs) such as cancer, T2D, hypertension, kidney disease, obesity, dental caries, and oxidative stress and have also shown antimicrobial activity (Ahmad et al., 2020; Singh & Rao, 2005; Talevi, 2021). Some recent studies found that stevia consumption can help to main weight, energy intake, and appetite (Stamataki et al., 2020; Stamataki, Scott, et al., 2020).

**FIGURE 1:**
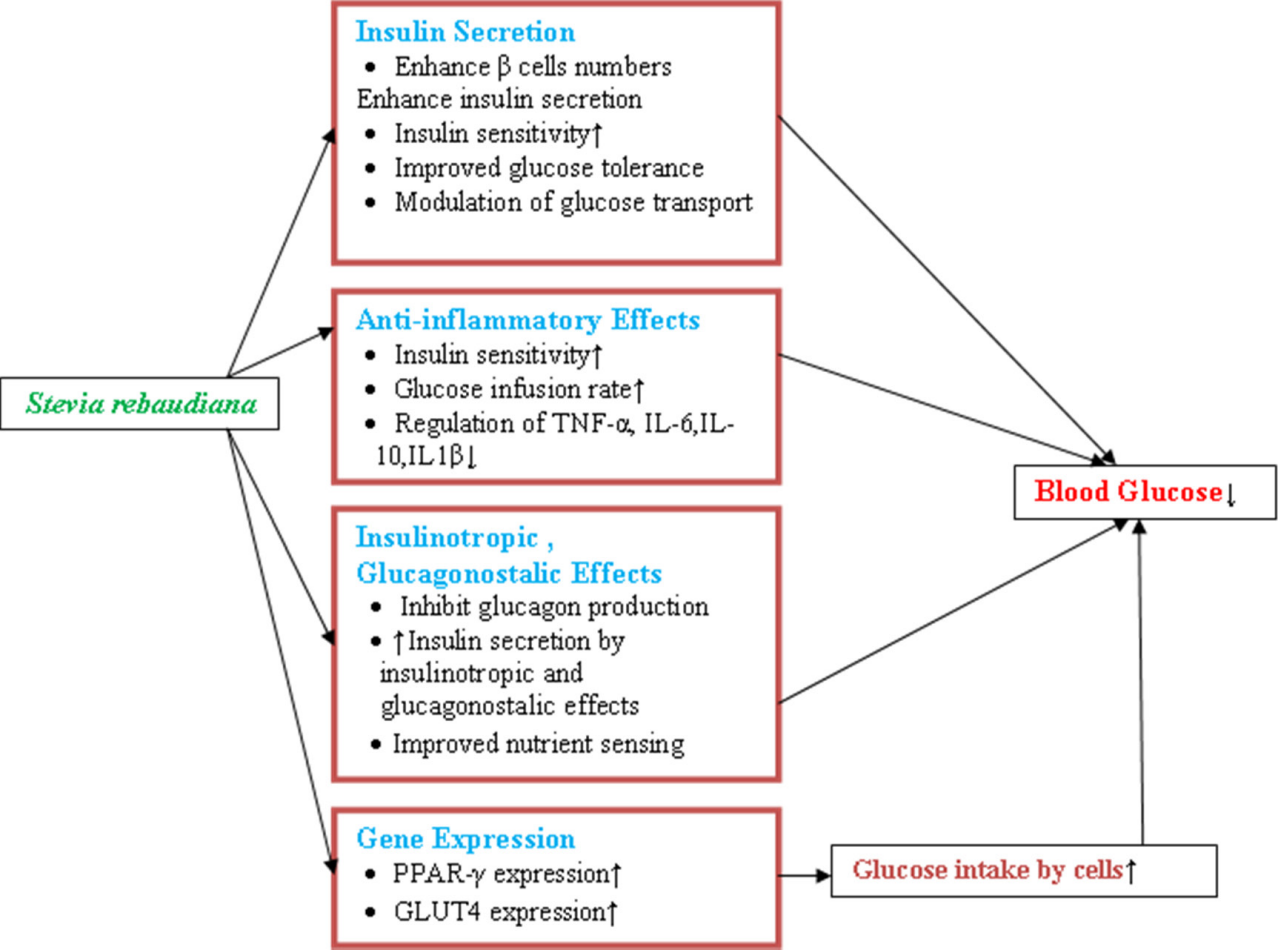
Actions of *Stevia rebaudiana* on blood glucose level

The prevalence of diabetes among people is increasing day by day. Stevia leaves possess antihyperglycemic, insulin‐mimetic, insulinotropic, and glucogonostatic properties, which play important role in the management of diabetes although there is limitation of proper randomized control trials (Bastaki, 2015). Recent evidence suggests that stevia stimulates the secretion of insulin by acting on beta cells of the pancreas and also shows antioxidant properties (Assaei et al., 2016; Massoumi et al., 2020). It also increases the expression of glucose transporter type (Glut) 4 gene, protein, and glucose uptake (Bhasker et al., 2015). Several studies concluded that stevia reduces blood glucose by up to 35% in humans (Chaves et al., 2018; Mayasari et al., 2018). Figure 1 shows mechanistic interaction of stevia leaves with diabetic and antidiabetic effect. The postprandial incremental area under the curve has been reduced after consuming stevia‐sweetened beverage compared with other sweetened beverages and did not alter the FBG in T2D subjects at chronic consumption of Rebaudioside (Anton et al., 2010; Maki et al., 2008). Diabetes is not only managed by the prescription of doctors but also nutrition counseling and education. Diet plays an important role in the management of diabetes (Mooradian, 2003).

Although several animal studies have reported an antihyperglycemic effect of stevia leaves, the overall impact of these studies has not investigated yet. Therefore, the present study was aimed at a systematic evaluation of the antidiabetic action of stevia leaf. A meta‐analysis was also performed to evaluate the effect of stevia on the blood glucose level.

## 2 METHODS

### 2.1 Protocol

This systematic review was undertaken to evaluate the effects of *S*. *rebaudiana* leaves on diabetes (animal studies) using the prespecified protocol for systematic reviews and meta‐analysis (PRISMA) (Moher et al., 2009).

### 2.2 Sources of information and literature search strategy

For this study, the effects of stevia leaves on diabetes were reviewed based on the publication date of articles before September 2020 in national and international databases, and the selected articles were collected by using the following databases: PubMed, Cochrane library (CENTRAL), and DOAJ. The literature search was conducted between September 19, 2020 and October 03, 2020. The following keywords and their possible combinations were used in the search: stevia, *S*. *rebaudiana*, sweet leaf, stevioside, diabetes, type‐1 diabetes, type‐2 diabetes, diabetes mellitus, blood sugar, blood glucose, serum glucose, and insulin. Both keywords and medical subject terms (MeSH) were included in the search strategy. The following search strategy was applied: (stevia OR stevia rebaudiana OR sweet leaf OR stevioside) AND (diabetes OR diabetes mellitus OR type 1 diabetes OR type 2 diabetes OR blood sugar OR blood glucose OR serum glucose) AND (rat OR mice OR murine; Table S1). Searches were limited to articles published in the English language.

### 2.3 Eligibility criteria

The selection criteria for this present study are following: (a) all kind of experimental study, (b) in vivo animal study (murine studies), (c) primary aim at evaluating the effects of stevia leaves on diabetes, (d) used appropriate controlled groups, (e) measured the BGL, and (f) published in the English language. Unrelated articles, articles conducted on human, observational studies, dissertation, unpublished work, reviews and meta‐analysis, abstracts, letter to the editor, in vitro or ex vivo model study, patient study, review articles, and editorial or protocol study were excluded.

### 2.4 Data collection and extraction

Firstly, on the basis of the title and abstracts, articles were screened. After that, articles which were relevant were screened following the inclusion and exclusion criteria. Initially, two independent reviewers screened the titles and abstracts of the selected publications to determine whether they would satisfy the selection criteria. Any disagreements between the reviewers about selection of articles were resolved through consensus or consultation with a third reviewer. The data collection form included questions on the publication year, study design, setting and country, animal species (Murine species), sex, route of administration of intervention, sample size, duration of doses administration, and methods of diabetes induction. The main indicators of diabetes condition are SGL, FBG, serum insulin, etc. But for the present study, we have utilized the BGL as an indicator of diabetes, as it is the most common variable used in several studies. We excluded the studies that did not provide sufficient information required for the present study.

### 2.5 Methodological quality assessment

The quality of the studies was measured using the standard from SYRCLE's risk of bias tool (Hooijmans et al., 2014) and CAMARADES checklist for study quality "Gold Standard Publication Checklist to Improve the Quality of Animal Studies," published by Radboud University Nijmegen Medical Center (Hooijmans et al., 2010).

### 2.6 Statistical analysis

We assessed the heterogeneity of the articles by reporting the *I* ^2^ (% residual variation due to heterogeneity) which measured the heterogeneity that caused fraction of variance and tau^2^ (method of moments estimates of between‐study variance) of the pooled estimate. Heterogeneity is measured to established whether studies are consistent. The range of *I^2^* statistics is from 0 to 100% (Higgins & Thompson, 2002). Studies were weighted according to an estimate of the standard mean difference (SMD), and a random‐effects model was used. The 95% confidence interval has been reported in a pooled analysis. Subgroup analysis was conducted if the analysis showed *p* ≤ .1 and *I* ^2^ > 50%. Subgroup analysis was conducted to explore the reasons and sources of heterogeneity. In this study, subgroup analysis was defined based on the duration of intervention. A funnel plot was also observed to identify the publication bias. Meta‐analysis was performed using Review Manager (RevMan) software version 5.4.

## 3 RESULT

### 3.1 Study selection

**FIGURE 2:**
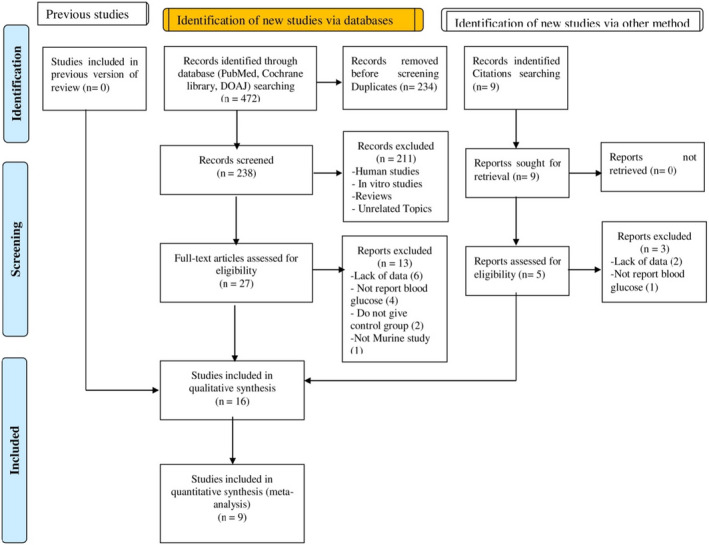
Flowchart of search strategy

A total of 481 studies were retrieved by searching in different electronic databases (PubMed, Cochrane library, and DOAJ) and references based on the titles and abstracts, of which 247 were unique. After screening these articles based on inclusion and exclusion criteria, 211 studies were excluded, and 32 articles were included for full‐text eligibility for detail screening. After the evaluation of these 32 studies, a total of 16 studies were excluded, and 16 studies (Abdel‐Aal et al., 2020; Ahmad & Ahmad, 2018; Akbarzadeh et al., 2015; Das et al., 2017; Ilić et al., 2017; Jeppesen et al., 2006; Kujur et al., 2010; Misra et al., 2011; Myint et al., 2020; Rashed et al., 2008; Rašković et al., 2008; Shivanna et al., 2013; Shukla et al., 2011; SUNANDA Singh et al., 2013; Suanarunsawat et al., 2004; Sumon et al., 2008) were enrolled in the present study for qualitative analysis, and 9 studies (Ahmad & Ahmad, 2018; Akbarzadeh et al., 2015; Das et al., 2017; Misra et al., 2011; Rashed et al., 2008; Shivanna et al., 2013; Shukla et al., 2011; SUNANDA Singh et al., 2013; Sumon et al., 2008) were selected for quantitative synthesis (Figure 2).

### 3.2 Characteristics of the studies

The characteristics of the selected studies were shown in Table 1. All selected studies evaluated the effects of stevia leaves on diabetes, and among these 16 studies, 9 studies were included in the statistical analysis due to providing appropriate data on BGL before and after the intervention. There was a considerable variation in the duration of intervention, age and sex of animal used, sample size, and species of animals. Among 16 studies, 4 studies were conducted on mice, and the rest on rats. Almost all articles reported the sex of animals (43.75% male) except 2 articles (Shivanna et al., 2013; SUNANDA Singh et al., 2013).

**TABLE 1:** Characteristics of included studies

Authors & year	Country	Duration (days)	Age (weeks)	Sex	Species	Diabetes induced	Rout of admn.	Freq/day	Sample size	Findings
Shruti et al. (2011)	India	21		Both	Rats	Alloxan	Orally	1	48	400 mg/kg ethanolic seed and leaf extracts reduced blood glucose levels in 52 and 43% at 3 h. 400 mg/kg dose was found more effective to reduce the blood glucose levels in a multidose study as compared to low range of doses.
Abdel‐aal et al. (2020)	Egypt	21		Male	Rats	STZ and nicotinamide	Stomach tube	1	40	Administration of stevia aqueous extract reduced fasting blood glucose (FBG) significantly (*p <* .001) compared to diabetic control stevia
Ahmad and Ahmad (2018)	Pakistan	56		Male	Rats	STZ	Orally	1	60	Blood glucose level (73.24%) and FBG (66.09%) decreased significantly (*p* < .05) in diabetic rats compared with diabetic and nondiabetic rats
Suanarunsawat et al. (2004)	Thailand	56		Male	Rats	STZ	Orally	1	–	*Stevia rebaudiana* (SR) significantly reduced the plasma glucose in diabetic rats
Jeppesen et al. (2006)	Denmark	28	20	Male	Rats	–	Orally	1	48	Stevia leaf with soy‐based dietary supplement has beneficial effect on diabetes and reduced plasma glucose 56% significantly
Kujur et al. (2010)	India	28		Both	Rats	Alloxan	Orally	1	42	Higher dose of aqueous and ethanol extract of stevia reduced mean blood glucose
Metha et al. (2011)	India	10		Both	Rats	Alloxan	Orally	1	70	Stevia decreased the blood glucose significantly but delayed
Myint et al. (2020)	China	42	5–6	Male	Mice	STZ	Orally	1	54	Steviol glycosides from stevia had positive effect in diabetic rats in blood glucose
Raskovic et al. (2008)	Serbia	14		Both	Rats	Alloxan	Orally	1	–	Stevioside significantly reduced glycemia with sodium salt
Rashed et al. (2008)	Bangladesh	21		Both	Rats	STZ	Orally	1	30	Extracts of stevia reduced blood glucose level 10–30% on 21 days at 3 ml/kg significantly
Shivanna et al. (2013)	India	35	12–13		Rats	STZ	Orally	1	80	Powder of stevia leaf reduced blood glucose level with increment of insulin level in diabetic rats
Singh et al. (2013)	India	21	28–35		Mice	Alloxan	Orally	1	28	Stevia leaf extract reduced sugar level of 39.8% in diabetic rats significantly
Das et al. (2017)	Bangladesh	56		Male	Mice	Alloxan	Orally	1	25	Stevia crystal reduced blood glucose significantly (*p* < .05)
Sumon et al. (2008)	Bangladesh	21	8–12	Both	Rats	STZ	Orally	1	30	Stevia reduced blood glucose level significantly (*p* < .05) with dose‐dependent relationship
Akbarzadeh et al. (2015)	Iran	30		Male	Rats	STZ	Orally	1	40	Fasting blood sugar reduced significantly among diabetic group with stevia administration
Ilić et al. (2017)	Serbia	10		Male	Mice	Alloxan	Orally	1	48	Stevioside from Stevia leaf prevented significant increase in glycemia values (*p* < .05)

### 3.3 Meta‐analysis

**FIGURE 3:**
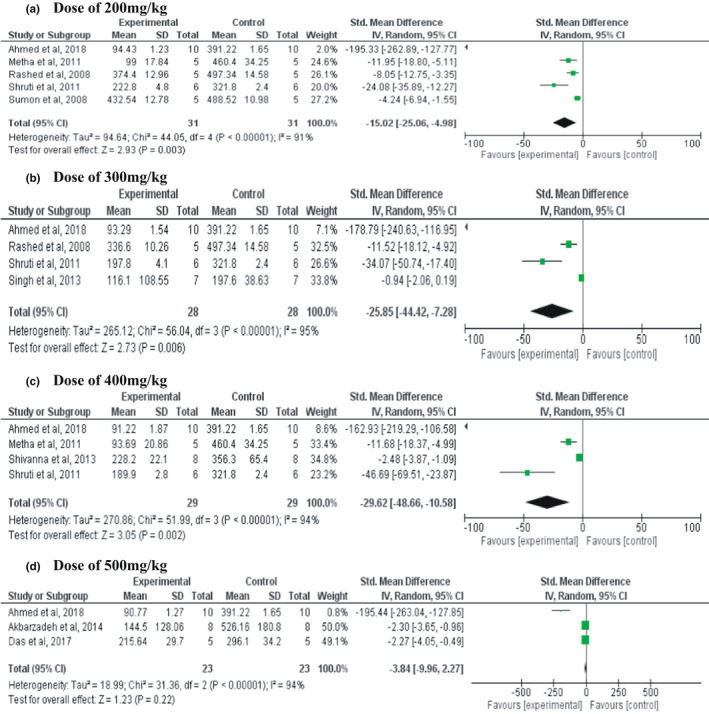
Forest plot of standard mean differences and 95% confidence interval (CI) of blood glucose level in animals treated with different doses of *Stevia rebaudiana* (200, 300, 400, and 500 mg/kg)

Figure 3 summarized the results of the outcome measures for the BGL. The overall meta‐analysis included 9 studies and four doses, i.e., 200, 300, 400, and 500 mg/kg of stevia leaves. In the meta‐analysis run for the dose, the 200 mg/kg dose included 5 studies, 300mg/kg dose included 4 studies, 400 mg/kg dose 4 studies, and 500 mg/kg dose 3 studies each (16 studies). The *pooled SMD* of 200, 300, and 400 mg/kg dose were −15.02 (*95% CI*: −25.06, −4.98), −25.85 (*95% CI*: −44.42, −7.28), and −29.62 (*95% CI*: −48.66, −10.58) and *p‐*value of them was *p* =.0003, 0.006 and 0.002 respectively. The results indicated that there was a significant difference in BGL between intervention and control group of animals. In contrast, the dose of 500 mg/kg showed that there was no significant difference in the *mean* where the *pooled SMD* and *p‐*value were −3.84 (−9.96, 2.27), 0.22. The meta‐analysis showed that the overall heterogeneity of the study was high and it was recorded to be (*I* ^2^ = 91%, *p* <.001) at 200 mg/kg, (*I* ^2^ = 95%, *p* < .001) at 300 mg/kg, (*I* ^2^ = 94%, *p* < .001) at 400 mg/kg, and (*I* ^2^ = 94%, *p* < .001) at 500 mg/kg.

### 3.4 Subgroup analysis

Based on the duration of intervention, subgroup analysis of the articles was performed to assess the source of heterogeneity in the included studies (Table 2). A significant beneficial effect of stevia leaves consumption in all doses among diabetic groups was observed after the subgroup analysis based on the duration of the intervention (≤14 days, 14–28 days, and >28 days). A significant difference between the groups of the duration of intervention was also found with respective *p‐*value < 0.001. However, the overall heterogeneity of subgroup analysis was still high (*I* ^2^ = 93% at 200 mg/kg, *I* ^2^ = 94% at 300 mg/kg, *I* ^2^ = 95% at 400 mg/kg and *I* ^2^ = 96% at 500 mg/kg).

**TABLE 2:** Subgroup analysis of the doses

Subgroup	200 mg			300 mg			400 mg			500 mg		
	SMD(95% CI)	*p*	*I* ^2^	SMD(95% CI)	*p*	*I* ^2^	SMD (95% CI)	*p*	*I* ^2^	SMD (95% CI)	*p*	*I* ^2^
≤14 days	−5.78 (−7.79, −3.76)	<.001	89	−7.63 (−10.52, −4.74)	<.001	92	−12.08 (−16.29, −7.86)	<.001	94	−6.06 (−10.33, −1.78)	<.006	95
14–28 days	−24.65 (−37.11, −12.18)	<.001	92	−23.68 (−40.32, −7.04)	.0006	95	−62.02 (−78.22, −45.81)	<.001	28	−70.77(−88.14, −53.4)	<.001	0
>28 days	−130.76 (−162.28, −99.24)	<.001	47	−131.94 (−159.2, −104.68)	<.001	27	−106.7 (−187.09, −26.3)	.009	97	−27.62 (−38.85, −16.38)	.009	97
Overall	−10.16 (−12.88, −7.44)	<.001	93	−11.87 (−15.12, −8.63)	<.001	94	−19.87 (−24.37, −15.37)	<.001	95	−3.71(−18.27, −9.14)	<.001	96

### 3.5 Methodological quality of studies

On the basis of CAMARADES quality items, the average point of these studies was 6 out of 10, and in case of SYRCLE's risk bias, the average point was 17 out of 21 characteristics. Most of the studies were not scored in some points such as calculation of sample size before the experiment, blinding, numbers and reasons for exclusion, number of dead animals, temperature of the body, and total number of animals included in statistical analysis (Tables S2 and S3).

## 4 DISCUSSION

Diabetes is a common metabolic disorder, and more than 800 plants have been reported that can be used to treat diabetes. Herbal medicine is an alternative medicine for treating diabetes if it can be served with safety and efficacy. The most used plant metabolite is flavonoids that have antidiabetic activity. The flavonoids *stevioside* from stevia leaves is one of the dietary polyphenols with strong antidiabetic and antioxidant activity (Casas‐Grajales et al., 2019; Elhassaneen, 2019; Gregersen et al., 2004). Stevia leaf reduced the blood glucose and controlled glucagon in diabetic patients by increasing the insulinogenic index (Gregersen et al., 2004). Many studies have reported the valuable actions of stevia leaf and its active compounds on metabolic disorders such as cardiovascular disease (CVD), hypertension, cancer, etc. (Kurek & Krejpcio, 2019). A study demonstrated that stevia could act as prebiotics as it is not absorbed in the upper intestine, which can be used in treating complications like constipation in chronic kidney disease (CKD), as well as other NCDs (Deniņa et al., 2014). A randomized controlled trial on human demonstrated that Rebaudioside A, an active component of stevia, could improve the blood parameters and have antidiabetic activity (Tey et al., 2017).

The present systematic review and meta‐analysis reported the antidiabetic activity of stevia leaf and its active components in animal models. A random‐effect model was used in this review for meta‐analysis, and the serum blood glucose was used as an outcome measurement. As outcome measures were presented in different doses and durations, we compared the forest plots of different doses and durations. A dose‐dependent relationship was observed among different doses of intervention. The pooled estimation of the SMD showed that glucose level was significantly altered between the intervention and control group. In our review, all doses except 500 mg showed significant result on lowering BGL (*p* < .05). This may be due to lack of more careful preclinical studies or may be much differences in the BGLs in control and experimental group in one study than the others. However, subgroup analysis of all doses based on duration of intervention showed significant results (*p* < .01). Although there is lack of human studies evaluating the effects of stevia on diabetes, a study showed that stevia significantly lowered glucose level in noninsulin‐dependent diabetes patients (Ritu & Nandini, 2016). A study reported that flavonoids consumption could reduce BGLs in a dose‐dependent manner among diabetes patients (Liu et al., 2014).

Insulin resistance is strongly associated with diabetes, and it can occur in pregnancy, cancer, obesity, sepsis, burn trauma, and starvation. Insulin resistance also triggers the risk of developing metabolic disorders (Houstis et al., 2006; Shahreen et al., 2012). Stevia reduces the activity of nuclear factor *k*‐light chain enhancer of activated β cells, which increases insulin activity to lower the glucose level and increase the body insulin sensitivity and glucose infusion rate (Wang et al., 2012). Another study observed that phenolic compounds of stevia (stevioside and steviol) could alone increase the secretion of insulin in intestinal (INS) pancreatic β‐cell that helps to reduce BGL without increasing the risk of hypoglycemia (Gregersen et al., 2004; Jeppesen et al., 2000). Stevia also has an effect on insulinotropic and glucagonostatic that also increases the glucose secretion by suppressing glucagon (Jeppesen et al., 2002). Consumption of stevia leaves not only increases blood insulin level but also suppresses or lowers gluconeogenesis and thus maintains the blood glucose levels. Stevia also could lower the interleukin‐6 (IL‐6), IL‐1, and TNF‐α and thus could help in lowering insulin resistance in patients with diabetes (Boonkaewwan & Burodom, 2013). Along with the antihyperglycemic effects of stevia, it also contributes in lipid metabolism, bile acid metabolism, amino acid metabolism, carbohydrates metabolism, and so on (Holvoet et al., 2015), and it was found that bile acid has an essential role in the regulation of glucose, energy, and lipid metabolism (Porez et al., 2012).

The effect of stevia leaf and its components on diabetes looks promising, although heterogeneity of this review was relatively high and so it is not possible to make a conclusion based on the pooled estimation. The subgroup analysis of the studies showed the source of heterogeneity. Subgroup analysis of these studies showed that all doses produce a statistically significant effect of stevia on diabetes, and the effect of stevia on diabetes was much significant at longer duration of intervention. The present systematic review and meta‐analysis evaluated the effect of stevia on diabetes from preclinical studies, because it is important to perform a systematic review to identify the impact of the other studies and also for generating a hypothesis for future clinical studies (Vesterinen et al., 2014).

To the best of our knowledge, this is first systematic review and meta‐analysis that addressed the effects of stevia leaves on diabetes in animal studies. However, there are several limitations that need to be considered. Our findings may be affected by publication bias, and numerous factors may influence the mechanism of stevia on diabetes.

This present review may also have several implications. On the basis of the available evidence, stevia leaves might be a safe choice for the treatment of diabetes based on duration of intervention, as our subgroup analysis showed that prevention of diabetes largely depends on the duration of intervention. However, some studies showed that stevia can cause cancer and infertility. So more clinical studies on human have been recommended to find out the safe dose of stevia supplement which can be used as an antidiabetic alternative medicine.

## 5 CONCLUSION

In this present systematic review and meta‐analysis, the effect of stevia and its active compounds on diabetes in the animal models has been observed, and it was confirmed that stevia has antidiabetic activity. The subgroup analysis also showed that the antihyperglycemic activity of stevia was higher at higher doses. Furthermore, our study recommended that more clinical trials with long‐term follow‐up studies are needed to investigate the antidiabetic effect of stevia, a typical seasonal medicinal herbs, abundantly grown in many parts of world (Japan, India, Nepal, Europe, and North America), as an alternative natural means in reducing blood glucose, and thus play a role in widespread prevention of diabetes.

## CONFLICT OF INTEREST

The authors have declared no conflict of interest.

## Supporting information

Table S1‐S3

## Data Availability

Data can be available upon request.
