# Supplementary material for: Effect of stevia leaves (Stevia rebaudiana Bertoni) on diabetes: A systematic review and meta‐analysis of preclinical studies
Source: Food Sci Nutr. 2022 Apr 24;10(9):2868–78. doi: 10.1002/fsn3.2904 (PMC9469865; doi:10.1002/fsn3.2904)
Supplement: Supplementary file 1 — Table S1‐S3 [file FSN3-10-2868-s001.docx]

Supplementary Table1: Search Strategy

| Database | Keywords | Total article |
| --- | --- | --- |
| PubMed | (Stevia OR stevia rebaudiana OR stevias OR sweet leaf OR sweet basils)AND (blood glucose OR serum glucose OR fasting glucose level OR diabetes OR diabetes mellitus OR type 1 diabetes mellitus OR type 2 diabetes mellitus) | 139 |
| CENTRAL | (Stevia OR stevia rebaudiana OR stevias OR sweet leaf OR sweet basils)AND (blood glucose OR serum glucose OR fasting glucose level OR diabetes OR diabetes mellitus OR type 1 diabetes mellitus OR type 2 diabetes mellitus) | 198 |
| DOAJ | (Stevia OR stevia rebaudiana OR stevias OR sweet leaf OR sweet basils)AND (blood glucose OR serum glucose OR fasting glucose level OR diabetes OR diabetes mellitus OR type 1 diabetes mellitus OR type 2 diabetes mellitus) | 135 |

Supplementary Table2: The CAMARADES quality items

| Study | 1 | 2 | 3 | 4 | 5 | 6 | 7 | 8 | 9 | 10 | Total |
| --- | --- | --- | --- | --- | --- | --- | --- | --- | --- | --- | --- |
| Shruti et al,2011 | Y | Y | Y |  |  |  | Y | Y |  | Y | 6 |
| Abdel-aal et al,2020 | Y | Y |  | Y |  |  | Y | Y | Y | Y | 7 |
| Ahmed et al, 2018 | Y | Y | Y |  |  |  | Y | Y | Y | Y | 7 |
| Suanarunsawat et al, 2004 | Y | Y |  |  |  |  |  | Y |  | Y | 4 |
| Jeppesen et al, 2006 | Y | Y |  |  |  |  | Y | Y |  | Y | 5 |
| Kujur et al, 2010 | Y | Y | Y |  |  |  | Y | Y |  | Y | 6 |
| Metha et al, 2011 | Y | Y | Y |  |  |  | Y | Y | Y | Y | 7 |
| Myint et al, 2020 | Y | Y |  |  |  |  | Y | Y | Y | Y | 6 |
| Raskovic et al, 2008 | Y | Y |  |  |  |  | Y |  |  | Y | 4 |
| Rashed et al, 2008 | Y | Y | Y |  |  |  |  | Y |  | Y | 5 |
| Shivanna et al, 2013 | Y | Y |  |  | Y |  | Y | Y |  | Y | 6 |
| Singh et al, 2013 | Y | Y |  |  |  |  | Y | Y |  | Y | 5 |
| Das et al, 2017 | Y | Y | Y |  |  |  | Y | Y | Y | Y | 7 |
| Sumon et al, 2008 | Y | Y | Y |  |  |  |  | Y |  | Y | 5 |
| Akbarzadeh et al, 2014 | Y | Y | Y |  |  |  | Y | Y | Y | Y | 7 |
| Ilic et al, 2017 | Y | Y |  |  |  |  | Y | Y | Y | Y | 6 |

(1) peer reviewed publication; (2) presence of randomization of subjects into treatment groups; (3) assessment of dose–response relationship; (4) blinded assessment of behavioural outcome; (5) monitoring of physiological parameters such as body temperature; (6) calculation of necessary sample size to achieve sufficient power; (7) statement of compliance with animal welfare regulations; (8) avoidance of anaesthetic agents with marked intrinsic neuroprotective properties (e.g., ketamine); (9) statement of potential conflict of interests; (10) use of a suitable animal model.

Supplementary Table 3: Quality assessment of included studies

| Study quality | Shruti | Abdel-aal | Ahmed | Suanarunsawat | Jeppesen | Kujur | Metha | Myint | Raskovic | Rashed | Shivanna | Sumon | Singh | Das | Akbarzadeh | IlIc |
| --- | --- | --- | --- | --- | --- | --- | --- | --- | --- | --- | --- | --- | --- | --- | --- | --- |
| Research question specified and clear? | Y | Y | Y | Y | Y | Y | Y | Y | Y | Y | Y | Y | Y | Y | Y | Y |
| Outcome measures relevant for AD research | Y | Y | Y | Y | Y | Y | Y | Y | Y | Y | Y | Y | Y | Y | Y | Y |
| Are the characteristics of study population clear? |  |  |  |  |  |  |  |  |  |  |  |  |  |  |  |  |
| Species | Y | Y | Y | Y | Y | Y | Y | Y | Y | Y | Y | Y | Y | Y | Y | Y |
| Background/generation | Y | Y | Y | Y | Y | Y | Y | Y | Y | Y | Y | Y | Y | Y | Y | Y |
| Sex | Y | Y | Y | Y | Y | Y | Y | Y | Y | Y | N | Y | N | Y | Y | Y |
| Age | N | N | N | N | Y | N | N | Y | N | N | Y | Y | Y | N | N | N |
| Presence and correct control group? | Y | Y | Y | Y | Y | Y | Y | Y | Y | Y | Y | Y | Y | Y | Y | Y |
| Where the groups similar at baseline (if not randomized think of weight and sex etc.)? | Y | Y | Y | Y | Y | Y | Y | Y | Y | Y | Y | Y | Y | Y | Y | Y |
| Is the experiment randomized? | Y | Y | Y | Y | Y | Y | Y | Y | Y | Y | Y | Y | Y | Y | Y | Y |
| Kind of supplement mentioned(stevia/steviaoside)? | Y | Y | Y | Y | Y | Y | Y | Y | Y | Y | Y | Y | Y | Y | Y | Y |
| Age when supplementation started mentioned? | N | N | N | N | Y | N | N | Y | N | N | Y | Y | N | N | N | N |
| Duration of supplementation clear and specified? | Y | Y | Y | Y | Y | Y | Y | Y | Y | Y | Y | Y | Y | Y | Y | Y |
| Amount of stevia mentioned | Y | Y | Y | Y | Y | Y | Y | Y | Y | Y | Y | Y | Y | Y | Y | Y |
| Administration route specified | Y | Y | Y | Y | Y | Y | Y | Y | Y | Y | Y | Y | Y | Y | Y | Y |
| Is the timing of the supplementation during the day specified and similar in both groups? | Y | Y | Y | Y | Y | Y | Y | Y | Y | Y | Y | Y | Y | Y | Y | Y |
| Methods used for outcome assessment the same in both groups? | Y | Y | Y | Y | Y | Y | Y | Y | Y | Y | Y | Y | Y | Y | Y | Y |
| Did report animals who died or were otherwise removed from the study | N | N | N | N | N | N | N | N | N | N | N | N | N | N | Y | N |
| Blinded outcome assessment? | N | Y | N | N | N | N | N | N | N | N | N | N | N | N | N | N |
| Was the outcome assessment randomized across the groups? | N | N | N | N | N | N | N | N | N | N | N | N | N | N | N | N |
| Total number of animals included in statistical analyses clear? | Y | Y | Y | Y | Y | Y | Y | Y | Y | Y | Y | Y | Y | Y | Y | Y |
| Age of sacrificing animals mentioned? | N | N | N | N | Y | N | N | Y | Y | N | Y | N | Y | N | N | N |
| Quality Score | 17 | 18 | 17 | 17 | 18 | 17 | 17 | 18 | 17 | 17 | 17 | 18 | 17 | 17 | 16 | 15 |

Y= filling the criteria, N= Not filling the criteria
